# Genome-wide analysis reflects novel 5-hydroxymethylcytosines implicated in diabetic nephropathy and the biomarker potential

**DOI:** 10.20517/evcna.2022.03

**Published:** 2022-03-24

**Authors:** Ying Yang, Chang Zeng, Kun Yang, Shaohua Xu, Zhou Zhang, Qinyun Cai, Chuan He, Wei Zhang, Song-Mei Liu

**Affiliations:** ^1^Department of Clinical Laboratory, Center for Gene Diagnosis & Program of Clinical Laboratory, Zhongnan Hospital of Wuhan University, 430071 Wuhan, Hubei, China.; ^2^Department of Preventive Medicine, Northwestern University Feinberg School of Medicine, Chicago, Illinois 60611, USA.; ^3^Department of Chemistry and the Howard Hughes Medical Institute, The University of Chicago, Chicago, Illinois 60611, USA.; ^#^Authors contributed equally.

**Keywords:** Type 2 diabetes, nephropathy, epigenetics, 5-hydroxymethylcytosine, cfDNA

## Abstract

**Aim:**

Diabetic nephropathy (DN) has become the most common cause of end-stage renal disease in most countries for patients with type 2 diabetes (T2D). Elucidating novel epigenetic contributors to DN can not only enhance our understanding of this complex disorder but also lay the foundation for developing more effective monitoring tools and preventive interventions in the future, thus contributing to our ultimate goal of improving patient care.

**Methods:**

5-hydroxymethylcytosines (5hmC)-Seal, a highly selective chemical labeling technique, was used to profile genome-wide 5hmC, a stable cytosine modification type marking gene activation, in circulating cell-free DNA (cfDNA) samples from a cohort of patients recruited at Zhongnan Hospital, including T2D patients with nephropathy (DN, *n* = 12), T2D patients with non-DN vascular complications (non-DN, *n* = 29), and T2D patients without any complication (controls, *n* = 14). Differential analysis was performed to find DN-associated 5hmC features, followed by the exploration of biomarker potential of 5hmC in cfDNA for DN using a machine learning approach.

**Results:**

Genome-wide analyses of 5hmC in cfDNA detected 427 and 336 differential 5hmC modifications associated with DN, compared with non-DN individuals and controls, and suggested relevant pathways such as NOD-like receptor signaling pathway and tyrosine metabolism. Our exploration using a machine learning approach revealed an exploratory model comprised of ten 5hmC genes showing the possibility to distinguish DN from non-DN individuals or controls.

**Conclusion:**

Genome-wide analysis suggests the possibility of exploiting novel 5hmC in patient-derived cfDNA as a non-invasive tool for monitoring DN in high-risk T2D patients in the future.

## INTRODUCTION

Diabetic nephropathy (DN) is one of the most common complications of type 2 diabetes (T2D) and a leading cause of end-stage renal disease globally^[1]^. Approximately 20-40% of T2D patients will ultimately develop nephropathic diseases, thus posing a significant risk for T2D patients^[2]^. Early detection and preventive intervention of DN has been limited due largely to the lack of a comprehensive understanding of its complex pathogenesis and effective biomarkers. Notably, conventional clinical markers to evaluate renal functions of DN, including serum creatinine, estimated glomerular filtration rate (eGFR), and urinary albumin, can be influenced by many factors^[3]^. Pathologically, the “gold standard” to diagnose DN has been percutaneous renal biopsy. However, various complications can be caused by the procedure, such as bleeding, pain, and infection^[4]^. Therefore, investigation of novel molecular contributors implicated in DN would not only enhance our understanding of this disease but also provide opportunities to develop more effective diagnostic and preventive approaches. Of particular interest to us are novel epigenetic modifications revealed in circulating cell-free DNA (cfDNA), a clinically convenient liquid biopsy, which may reflect systematic changes in the body during pathogenesis^[5]^.

Particularly, epigenetic modifications are gene regulatory elements that sit between phenotypes and genotypes^[3]^. The most-investigated epigenetic modification is DNA methylation, i.e., 5-methylcytosine (5mC), which has been implicated in normal physiological processes and pathogenesis. The regulation of DNA methylation *in vivo* is a dynamic process. The ten-eleven translocation enzymes can oxidize 5mC into 5-hydroxymethylcytosine (5hmC), 5-formylcytosine, and 5-carboxylcytosine under an active demethylation process^[6]^. Unlike other demethylated products of 5mC, 5hmC is relatively abundant and biochemically stable in the human genome. Previous studies have confirmed that the 5hmC modifications show a distinct genomic distribution and gene regulatory role from 5mC^[7]^ and have been implicated in a variety of diseases. Notably, recent studies have begun to demonstrate an association of altered 5hmC with diabetes-related conditions such as hyperglycemia^[8]^.

Technically, the widely used bisulfite conversion-based epigenomic profiling techniques, although offering opportunities of profiling genome-wide cytosine modifications, cannot distinguish 5hmC from 5mC^[9]^. Therefore, to investigate whether the 5hmC modifications are implicated in DN, we utilized the 5hmC-Seal technique^[10]^, a highly sensitive chemical labeling technique for genome-wide profiling of 5hmC, and next-generation sequencing (NGS), in cfDNA samples derived from a cohort of T2D patients with and without nephropathy. The 5hmC-Seal technique has been systematically validated using spike-in controls and serial DNA inputs by our team and other groups as a reliable approach for biomarker discovery^[9,11-15]^ using limited clinical biospecimens, e.g., as low as a few nanograms of cfDNA that can be conveniently isolated from 1-2 mL of plasma^[11]^. Therefore, the 5hmC-Seal technique has a technical advantage, especially suitable for future clinical implementation of cfDNA-based non-invasive tools for disease diagnosis, prognosis, and surveillance. Furthermore, our previous genome-wide analyses of 5hmC in cfDNA suggested a link between altered 5hmC and T2D-associated vascular complications in general^[16]^. For example, the 5hmC-based signatures in cfDNA were shown to have the potential to distinguish T2D patients with multiple vascular complications from those with single vascular complications^[16]^, as well as between T2D patients who developed diabetic retinopathy and those who did not^[17]^. However, whether there are specific 5hmC changes implicated in DN has not been investigated yet. 

**Figure 1:**
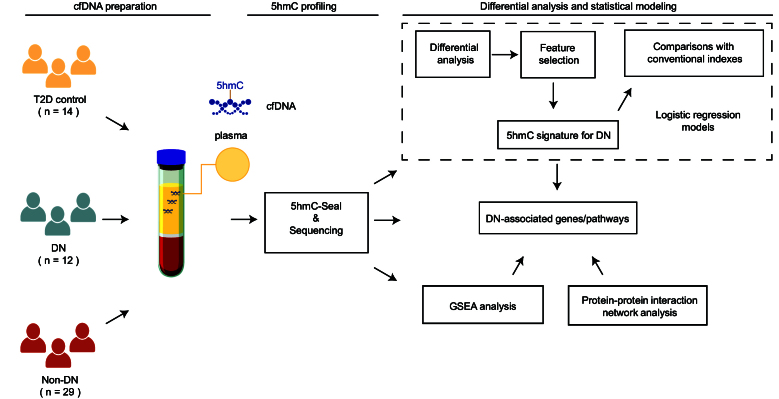
Study design. In total, 55 patients with type 2 diabetes (T2D), including 12 patients with diabetic nephropathy (DN), 29 patients with non-DN complications (Non-DN), and 14 controls (CTRL), were profiled for genome-wide 5-hydroxymethylcytosines (5hmC) using the 5hmC-Seal technique and next-generation sequencing, followed by differential analysis, gene set enrichment analysis (GSEA), protein-protein interaction network analysis, feature selection, and modeling to inform biological insights and evaluate biomarker potential.

Specifically, in the current study [Figure 1], we profiled genome-wide 5hmC in cfDNA samples derived from a cohort of 55 patients with T2D using the 5hmC-Seal technique and NGS. Differential analysis was performed to identify DN-associated modified genes as well as involved pathways. To investigate the feasibility of future biomarker studies targeting 5hmC for DN, we also explored the distinguishing capacity of 5hmC for DN by summarizing the genome-wide 5hmC profiles through feature selection using a machine-learning approach. Findings from this study enhance our understanding of DN-associated epigenetic changes and involved pathways, and provide the foundation for developing more effective and non-invasive tools for DN monitoring and preventive intervention in the future.

## METHODS

### Study populations

In total, 55 patients with T2D, including 12 patients with DN, 29 patients with non-DN complications (i.e., macrovascular complications, neuropathy, and retinopathy), and 14 sex- and age-matched T2D controls without complications, were recruited at Zhongnan Hospital of Wuhan University, China. Patients were diagnosed according to the 2017 Standards of Medical Care in Diabetes of the American Diabetes Association^[18]^. All study participants were excluded for other kidney diseases. Clinical variables were collected from the medical records following a standard protocol. Fasting plasma samples (~ 2 mL/patient) were collected the next morning after hospital admission. This study was approved by the Medical Ethics Committee of Zhongnan Hospital of Wuhan University (2019069). Informed consent was obtained from each participant.

### Laboratory measurements

Laboratory measurements were performed at Zhongnan Hospital for the current study. Kidney function parameters (creatinine, urea nitrogen, uric acid, and eGFR)^[19]^ and serum glucose were examined by the AU5800 Chemistry Analyzer (Beckman). The HA-8160 Glycohemoglobin Analyzer was used to measure blood glycated hemoglobin (HbA1c). Serum insulin was assayed by the i4000SR Immunology Analyzer (Abbott Laboratories). 

### Preparation of cfDNA samples, 5hmC-Seal assay, and data processing

Details about the preparation of circulating cfDNA samples, 5hmC-Seal library construction, sequencing, and data processing are described in our previous publications^[10,11,20]^. Briefly, plasma samples were separated and stored at - 80 °C after centrifuging twice at 1350 × *g* for 12 min and 13,500 × *g* for 5 min. cfDNA was extracted from the plasma using the Circulating Nucleic Acid Kit (Qiagen) and the concentration of cfDNA was examined using the Qubit High Sensitivity dsDNA Assay (Invitrogen) according to the manufacturers’ instructions. The 5hmC-Seal library construction and NGS were performed at the Innovation Center for Genomics, Peking University (Beijing, China). Briefly, each cfDNA sample was first prepared and ligated with adaptors. Next, the T4 bacteriophage enzyme β-glucosyltransferase was used to transfer an engineered glucose moiety containing an azide-group to 5hmC in duplex DNA. A biotin tag was then installed onto the azide group using Click chemistry, followed by capturing of 5hmC-containing DNA fragments using avidin beads. The 5hmC-Seal library for each cfDNA sample was then constructed through PCR amplification, followed by paired-end sequencing (PE39) with the Illumina NextSeq 500 platform. On average, ~ 23 million unique reads per cfDNA sample were obtained from NGS. According to our previous studies^[11-13,16,17]^, 5hmC profiles are more abundant in gene bodies and exonic regions relative to their flanking regions and depleted at the transcription start sites. Therefore, well-annotated gene bodies provided by GENCODE (hg19)^[21]^ were our primary targets to summarize the 5hmC-Seal data by counting the sequencing reads using feature Counts^[22]^. The principal components analysis (PCA) was conducted to explore the potential confounding factors in global 5hmC data. The kidney-derived histone modification marks for enhancers, i.e., H3K4me3 and H3K27ac, were obtained from the Roadmap Epigenomics Project^[23]^ to help provide biological insights. 

### Identifying DN-associated 5hmC signature in cfDNA

Multivariable logistic regression models were used to identify gene bodies containing differential 5hmC levels (i.e., normalized read counts) between DN patients and T2D controls, as well as between DN and non-DN patients. Although not the focus of the current study, we also performed differential analysis between T2D controls and patients with non-DN complications for comparison. Adjusted covariates included age and sex. To evaluate potential protein–protein interaction (PPI) networks, those genes showing a trend of differential modifications (Wald test *P *< 0.01 and fold change > 10%) between diagnosis classes, e.g., DN* vs.* controls, were supplied to the *stringApp* from Cytoscape^[24,25] ^with the default parameters based on the STRING database (confidence score > 0.8 and maximum additional interactor = 50) with linker genes allowed^[26]^. Hubs of the PPI networks were estimated based on the measurement of betweenness centrality, which represents the magnitude of influence a component gene has over the flow of information in a gene network^[24]^. Moreover, because of the limited sample size, instead of evaluating pathways among individual genes, Gene Set Enrichment Analysis (GSEA)^[27]^ was used to explore the functional relevance of canonical pathways in the whole-genome 5hmC data between diagnosis classes, e.g., DN *vs.* controls, using the clusterProfiler tool(v4.0)^[28]^. Specifically, over-/under-represented pathways maintained in the Kyoto Encyclopedia of Genes and Genomes (KEGG)^[29]^ database (≥ 15 genes and false discovery rate < 5%) and normalized enrichment score were obtained from GSEA. 

### Summarization of a 5hmC-based epigenetic score for DN

To evaluate whether a cfDNA-based score with potential diagnostic value could be summarized from the genome-wide 5hmC data, those genes that showed a trend of differential 5hmC between DN and controls or DN and non-DN complications, but not between non-DN complications and controls, were further selected to explore a signature panel by applying the elastic net regularization^[30]^ on the multivariable logistic regression models. To improve modeling efficiency, we filtered out most uninformative gene bodies (i.e., *P* > 0.05) before feature selection. Component genes of the exploratory model were selected if they were consistently present (100%) in 100 iterations using repeated two-fold cross-validation to differentiate between DN and controls. A weighted score to summarize the genome-wide 5hmC for each individual was computed as follows:

**Figure:**
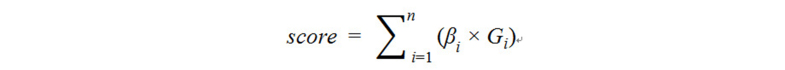


where *G_i_* represents the normalized read counts of the *ith* gene body and *β_i_* represents its regression coefficient, following our previous publications^[11,12,16,17]^. The area under the receiver operator characteristic curve (AUROC) was used to demonstrate model performance. The optimal score cutoffs for the AUROCs were determined by the score that maximized the Youden index, and the corresponding sensitivity and specificity were estimated.

### Comparison between the 5hmC-based score for DN with conventional clinical variables or risk factors

To compare the performance of the 5hmC-based scores for DN relative to various clinical variables, univariable logistic regression models for available clinical variables were examined as follows:

**Figure:**
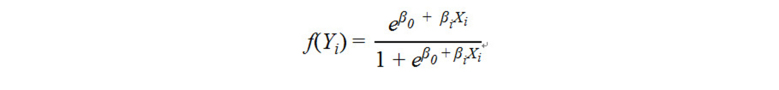


where *Y_i_* represents binary diagnosis classes (i.e., DN *vs.* non-DN/controls or DN *vs.* non-DN). *X_i_* represents age, sex, or each of the clinical variables body mass index (BMI), smoking history, drinking history, glucose, HbA1c, insulin, creatinine, uric acid, urea nitrogen and eGFR. The predicted probabilities of the univariable logistic regression models were used for assessing classification performance, i.e., DN *vs.* non-DN/controls or DN *vs.* non-DN, via the AUROC. Sensitivity and specificity at the cutoff that maximized the Youden index were estimated for each variable. 

## RESULTS

### Clinical and demographic characteristics of the study participants

Table 1 shows the clinical and demographic characteristics of the 55 study participants. Overall, there were no significant differences regarding major demographic and clinical variables between patient groups. There were comparable distributions of potential confounders for epigenetic modifications between patient groups, such as baseline BMI and sex (*P* > 0.05). Notably, differences in age at the time of blood collection were observed between T2D controls and DN patients. Therefore, age was used as a covariate in downstream differential analysis when comparing between diagnosis groups (e.g., DN *vs.* controls). Moreover, in total, 24 patients used insulin treatment and 27 patients used oral glucose-lowering medications, showing no significant disparity regarding medication treatment between different diagnosis groups (*P* > 0.05). 

**Table 1:** Demographical and clinical characteristics of the study participants

**Clinical variable**	**T2D control (*n *= 14)**	**DN (*n *= 12)**	**Non-DN (*n *= 29)**	**p^A^**	**p^B^**	**p^C^**
Age (year)	47.1 ± 9.7	56.8 ± 14.5	57.7 ± 8.1	0.08^a^	0^a^	0.71^a^
Sex (male/female)	10/4	9/3	13/16	0.52^b^	0.14^b^	0.25^b^
BMI (kg/m^2^)	24.9 ± 3.7	25.4 ± 2.3	25.7 ± 4.1	0.49^a^	0.62^a^	0.99^a^
Smoking (Yes/No)	6/8	7/5	5/24	0.69^b^	0.15^b^	0.02^b^
Drinking (Yes/No)	2/12	4/8	4/25	0.5^b^	1^b^	0.32^b^
T2D duration (year)	3.7 ± 3.9	4.5 ± 6.4	6.9 ± 6.3	0.7^a^	0.14^a^	0.08^a^
SBP (mmHg)	123.6 ± 9.4	135.4 ± 20.2	133.9 ± 19.2	0.17^a^	0.09^a^	0.82^a^
DBP (mmHg)	78.3 ± 7.9	82.1 ± 10.6	77.8 ± 10.6	0.28^a^	0.47^a^	0.13^a^
Glucose (mmol/L)	11.5 ± 3.3	9.5 ± 3.5	8.5 ± 3.1	0.13^a^	0.01^a^	0.46^a^
HbA1c (%)	9.1 ± 1.8	7.9 ± 2.2	8.4 ± 1.9	0.17^a^	0.33^a^	0.42^a^
Insulin (μU/mL)	10.3 ± 6.0	7.4 ± 7.1	9.5 ± 7.0	0.28^a^	0.66^a^	0.28^a^
Kidney Function:						
Urea nitrogen (mmol/L)	5.8 ± 1.3	5.7 ± 1.8	5.9 ± 1.7	0.78^a^	0.83^a^	0.69^a^
Creatinine (μmol/L)	60.3 ± 13.9	78.8 ± 31	63.1 ± 18.2	0.15^a^	0.86^a^	0.14^a^
Uric acid (μmol/L)	283.6 ± 51.9	319.7 ± 99.1	291.1 ± 81.9	0.4^a^	0.93^a^	0.51^a^
eGFR (mL/min/1.73 m^2^)	111.6 ± 12.2	89.2 ± 28.4	98.5 ± 13.4	0.02^a^	0.01^a^	0.24^a^
Medication (Yes/No)						
Insulin treatment	6/8	3/9	15/13	0.59^b^	0.74^b^	0.19^b^
Oral glucose-lowering medicine	6/8	6/6	15/13	1^b^	0.74^b^	1^b^

^A^Comparison between T2D control (CTRL) and Nephropathy (DN); ^B^comparison between T2D control (CTRL) and patients with non-nephropathy complications (Non-DN); ^C^comparison between DN and Non-DN; ^a^ Wilcoxon test; ^b^chi-square test. DN: diabetic nephropathy; BMI: body mass index; T2D: Type 2 diabetes; SBP: systolic blood pressure; DBP: diastolic blood pressure; HbA1c: glycated hemoglobin; eGFR: estimated glomerular filtration rate.

### Overview of the genome-wide 5hmC profiles in cfDNA

Consistent with our observations in the cfDNA samples from other studies^[13,15]^, the distribution of genome-wide 5hmC was also more abundant in gene bodies and exonic regions relative to their flanking regions and the transcription start sites [Supplementary Figure 1A], supporting our focus on gene bodies in this proof-of-concept study. Moreover, PCA demonstrated no significant correlations between 5hmC and potential confounders, including sex and age [Supplementary Figure 1B and C]. In addition, we observed a trend of increased genome-wide 5hmC modification levels on kidney-derived enhancer marks: H3K4me1, across controls, patients with non-DN complications, and patients with DN (*P*-trend = 0.049).

### Differentially modified genes associated with DN and the PPI network analysis

**Figure 2:**
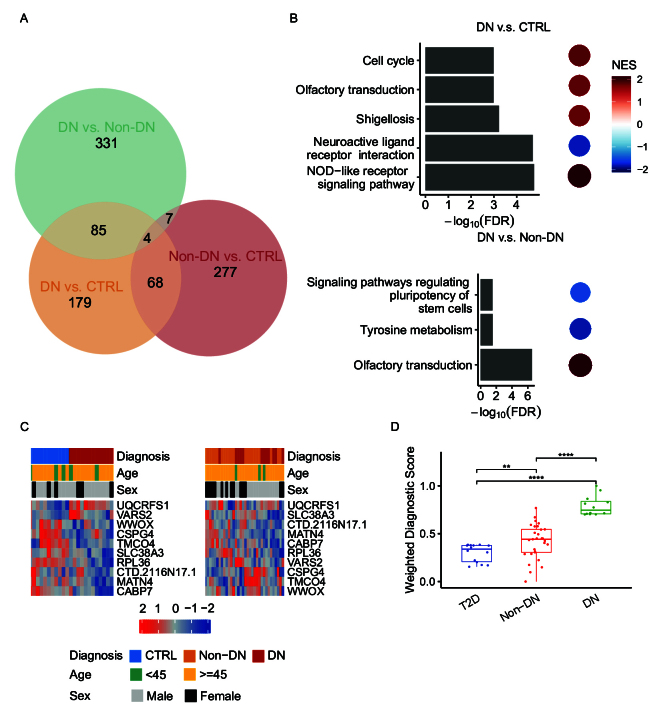
Novel 5hmC modifications implicated in diabetic nephropathy. Genome-wide analysis of the 5hmC-Seal data in patient-derived cfDNA reflected novel epigenetic modifications implicated in diabetic nephropathy (DN). (A)The Venn diagram shows differentially modified gene bodies specific to DN. (B)KEGG pathways significantly over-/under-represented in DN patients relative to either controls (CTRL) or non-DN patients (Non-DN) were identified from the GSEA. (C) The exploratory model comprised of ten component genes could distinguish DN from CTRL, as well as DN from Non-DN. (D) The 5hmC scores computed with the ten-gene exploratory model for DN were significantly different between DN and CRTL/Non-DN. Statistical significance (*t*-test): ns, *P* > 0.05; ** *P* ≤ 0.01; **** P ≤ 0.0001. KEGG: Kyoto Encyclopedia of Genes and Genomes; GSEA: Gene set enrichment analysis; NES: Normalized enrichment score.

In total, 336 genes were detected to show a trend of differential modification between T2D controls and patients with DN (Wald test *P* < 0.05), among which 271 genes had a fold change of at least 10% (Supplementary Table 1 and Supplementary Figure 1D). In comparison, 427 genes were found to be differentially modified between patients with DN and patients with non-DN complications (Wald test *P* < 0.05), among which 250 genes had a fold change of at least 10%, indicating the presence of 5hmC signatures specific to DN complications [Figure 2A, Supplementary Table 1, and Supplementary Figure 1E]. Genes with a more stringent cutoff (Wald test *P* < 0.01 and fold change > 10%) were further evaluated for PPI networks to explore potential biological connections with relevant functions. Notably, the PPI network analysis implicated those genes showing differential modifications between DN and T2D controls in hub genes relevant to kidney diseases [Supplementary Figure 2]. For example, SMARCA5*, *a member of the SWI/SNF-related matrix-associated action-dependent regulator of chromatin subfamily A as well as a differentially modified gene, was found to be connected with biomarkers for diabetic kidney disease^[31]^. *RUVBL1*, which encodes RuvB like AAA ATPase 1, is connected with certain differential genes (e.g., *COMMD2* and *GPS1*), and its deletion could lead to acute kidney injury in mice^[32]^. In contrast, the PPI network analysis based on a list of genes showing differential 5hmC between DN and non-DN patients also identified connections with hub genes implicated in DN [Supplementary Figure 3]. For example, signal transducer and activator of transcription 1 (STAT1) are connected with certain differential genes (e.g., *MX1*, *IFI44L*, and *IFIH1*), and its activation was shown to cause cell apoptosis and renal fibrosis, thus being implicated in DN^[33]^.

### GSEA implicating pathways differentially modified in patients with DN

The GSEA results reveal over- or under-representation of certain canonical pathways in patients with DN relative to controls, such as the NOD-like receptor signaling pathway, neuroactive ligand-receptor interaction, platelet activation, tyrosine metabolism, and necroptosis [Figure 2B and Supplementary Table 2]. Several core genes that contributed to the over- or under-representation of these pathways were also differentially modified between DN and T2D controls, including *CXCL1* and *PKN2* in the NOD-like receptor signaling pathway; *PYY*, *GRM*, *EDN2*, *GCGR,* and *MLN* in neuroactive ligand-receptor interaction; and *IL1B* in necroptosis [Supplementary Table 2]. In addition, the GSEA results between DN patients and patients with non-DN complications indicate significant over- or under-representation of KEGG pathways such as tyrosine metabolism, olfactory transduction, and signaling pathways regulating pluripotency of stem cells [Figure 2B and Supplementary Table 2], although they are not differentially modified at the single-gene level, likely due to the small sample size. Interestingly, several over-represented pathways between DN and controls/non-DN patients are known to be associated with DN or kidney-related diseases, such as the NOD-like receptor signaling pathway and tyrosine metabolism^[34,35]^. 

### Summarization of 5hmC-based epigenetic score for DN

An exploratory model comprised of ten genes (i.e., *UQCRFS1*, *VARS2*, *WWOX*, *CSPG4*, *TMCO4*, *SLC38A3*, *RPL36*, *CTD.2116N17.1*, *MATN4*, and *CABP7*) was identified using the elastic net regularization and multivariable logistic regression models for distinguishing DN from T2D controls [Figure 2C]. Of note, the 5hmC scores were significantly different between patients with DN and controls, as well as between patients with DN and those with non-DN complications [Figure 2D] (*t*-test, *P* < 0.01). When using the 5hmC score as the only predictor, the AUROC results show 100% sensitivity and 97% specificity to classify DN and non-DN complications, in addition to the performance of distinguishing patients with DN from controls [Table 2]. 

**Table 2:** Biomarker potential of the 5hmC model and comparisons with clinical variables

	**T2D control *vs*. DN**			**Non-DN *vs*. DN**		
Clinical variable/model	Sensitivity	Specificity	AUROC	Sensitivity	Specificity	AUROC
Age (year)	0.83	0.50	0.70	0.42	0.83	0.54
Sex (male/female)	0.75	0.29	0.52	0.75	0.55	0.65
BMI (kg/m^2^)	1.00	0.36	0.58	1.00	0.24	0.50
Smoking (yes/no)	0.58	0.57	0.58	0.58	0.83	0.71
Drinking (yes/no)	0.33	0.86	0.60	0.33	0.86	0.60
Glucose (mmol/L)	0.73	0.64	0.68	0.55	0.68	0.58
HbA1c (%)	0.55	0.86	0.67	0.55	0.85	0.59
Insulin (μU/mL)	0.83	0.62	0.69	0.67	0.71	0.66
Creatinine (μmol/L)	0.36	1.00	0.68	0.36	0.93	0.65
Uric acid (μmol/L)	0.64	0.71	0.60	0.73	0.48	0.57
Urea nitrogen (mmol/L)	0.45	0.86	0.54	0.45	0.72	0.54
eGFR (mL/min/1.73 m^2^)	0.64	1.00	0.78	0.64	0.79	0.62
5hmC model	1.00	1.00	1.00	1.00	0.97	0.98

T2D: Type 2 diabetes; DN: diabetic nephropathy; AUROC: the area under the receiver operator characteristic curve. BMI: body mass index; HbA1c: glycated hemoglobin; eGFR: estimated glomerular filtration rate.

We compared the sensitivity and specificity of various clinical variables in our cohort for distinguishing DN from T2D controls or patients with non-DN complications [Table 2]. Notably, logistic regression results indicate that the 5hmC scores in general outperformed age, sex, and various conventional clinical variables, including clinical variables of kidney functions, featuring greater AUROCs and higher sensitivity/specificity [Table 2]. For example, the 5hmC scores significantly outperformed the eGFR in differentiating between patients with DN and controls (AUROC, 100% *vs.* 78%) as well as between DN and non-DN complications (AUROC, 98%* vs.* 62%).

## DISCUSSION

Enhancing our understanding of the molecular contributors to DN pathogenesis would provide opportunities for developing more effective clinical tools to prevent and manage this complication. Equipped with the highly sensitive 5hmC-Seal technique, we sought to investigate DN-associated 5hmC in patient-derived cfDNA using a cohort of T2D patients with and without DN. Genome-wide analysis of 5hmC indicated there existed differential 5hmC modifications and over-/under-represented pathways in cfDNA that provided links between 5hmC signatures for DN and relevant pathways/genes. Besides previously implicated pathways and genes in DN or kidney disease, such as the NOD-like receptor signaling pathway and *CXCL1* of the inflammasome family^[34,36]^, interestingly, our identified DN-associated 5hmC signatures were also shown to be connected with PPI hubs relevant to kidney disease and the pathogenesis of DN^[32,33]^, thus reflecting the DN relevance of the 5hmC profiles in patient-derived cfDNA. Additionally, certain significant pathways such as Fc gamma R-mediated phagocytosis and natural killer cell-mediated cytotoxicity were found to be enriched in those genes dysregulated in DN from a meta-analysis of mouse microarray data^[37]^, lending further support for the existence of biological links between the 5hmC landscape reflected in DN patient-derived cfDNA and the underlying pathogenesis. 

One important question about the cfDNA-based methods is whether the patient-derived cfDNA samples reflect the target tissue. Our genome-wide scan examining co-localization of the 5hmC-Seal reads and kidney-derived enhancer markers demonstrated a trend of increased modification levels between T2D controls and DN patients, suggesting the contribution of the target tissue (i.e., kidney) to the 5hmC profiles in DN patients. The current tissue-derived histone modifications, however, included only two individuals from the Roadmap Epigenomics Project; with the availability of more reference epigenomes in the future, a more comprehensive evaluation would provide more insights into the relative proportions of cfDNA sources in patients with DN.

Considering that cfDNA could reflect the systematic and dynamic physiological condition of the patient, our findings targeting novel epigenetic information in cfDNA could provide the foundation for developing a convenient clinical tool for the care of T2D patients. Therefore, besides differential analysis, we also sought to evaluate the possibility of summarizing the genome-wide 5hmC profiles in cfDNA into an epigenetic score with biomarker potential. Particularly, findings from the feature selection based on machine learning and modeling in the current study provided promising results for the future development of cfDNA-based diagnostic or monitoring tools for DN. In particular, although limited by the sample size, the 5hmC-based exploratory model for DN showed a consistent trend of outperformance over various conventional clinical indices for diabetic complications, especially those related to kidney functions, thus supporting the potential advantage of utilizing the 5hmC features in cfDNA as a novel biomarker for DN. Moreover, because the 5hmC-Seal technique can provide the genome-wide distribution of 5hmC in a single test, it is possible to integrate the DN-associated model with models for other diabetic complications to develop a comprehensive tool for routine care of patients with T2D in the future.

We are aware of several limitations in the current study. Firstly, the sample size is relatively small. Although our primary goal was to demonstrate the relevance of 5hmC to DN and the feasibility of using novel epigenetics and non-invasive liquid biopsy to develop management tools for DN in the future, future larger scale investigations studies will be necessary to provide a more comprehensive picture of the epigenetic landscape of DN or DN-associated pathways. Secondly, also limited by the current sample size, our modeling of 5hmC for their biomarker value was preliminary using a single cohort. Although testing using patients with and without DN helped us evaluate the biological relevance of the identified 5hmC features, future investigations involving more independent samples for both training and independent validation will be necessary to develop a clinically useful model based on 5hmC in cfDNA. Thirdly, the current study only focused on the 5hmC modification over genic regions; because the functional relevance of genic regions was better annotated and established, it would be interesting to extend the 5hmC analysis to other genomic regions, such as long non-coding RNA^[38]^ and enhancer markers as well as co-regulation analysis between 5hmC and gene expression in the future. Finally, future studies that expand to other populations and ethnic backgrounds will provide insights into any population-specific epigenetic modifications associated with DN, because of the long-appreciated baseline differences in epigenetic modifications across human populations^[39]^.

In conclusion, novel 5hmC modifications detected in patient-derived cfDNA samples were found to be implicated in DN. The 5hmC-Seal technique implemented with cfDNA holds promise for the future development of a non-invasive, clinically convenient tool for early detection of DN in high-risk T2D patients, thus contributing to the ultimate goal of improving clinical outcomes through personalized preventive intervention and/or treatment. 

## DECLARATIONS

### Availability of data and materials

The 5hmC-Seal sequencing data and related metadata have been deposited into the NCBI Gene Expression Omnibus database (Accession No. GSE186021).

### Copyright

© The Author(s) 2022.
