# Supplementary material for: Genome-wide analysis reflects novel 5-hydroxymethylcytosines implicated in diabetic nephropathy and the biomarker potential
Source: Extracell Vesicles Circ Nucl Acids. 2022 Mar 24;3(1):49–60. doi: 10.20517/evcna.2022.03 (PMC8950161; doi:10.20517/evcna.2022.03)
Supplement: Supplementary file 1 [file evcna-3-1-49-SupplementaryMaterials.pdf]

ocu

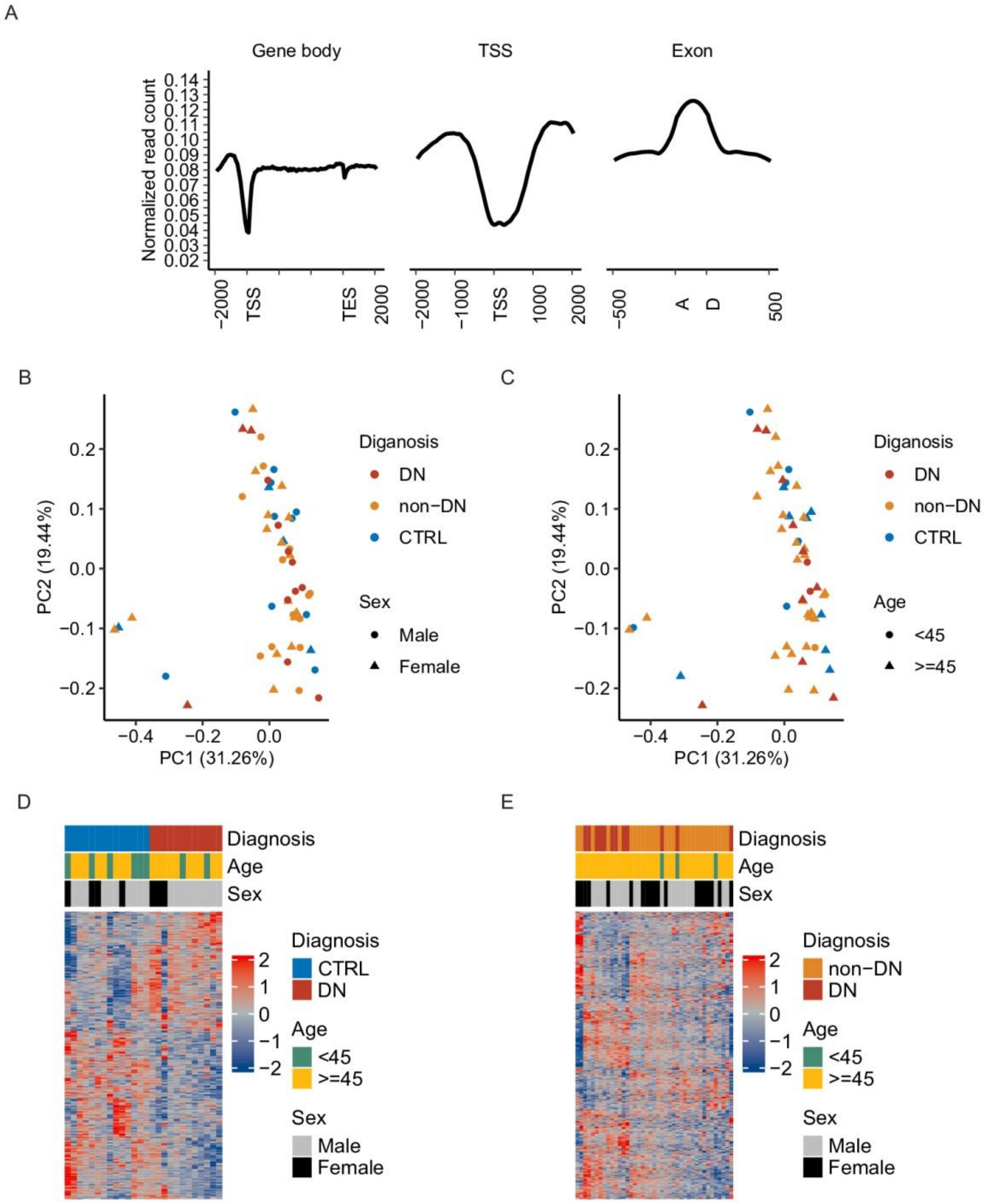

Supplementary Figure 1. Genome-wide distributions of 5hmC in cfDNA samples. (A) The 5hmC modifications are distinctly distributed across various genomic features. The read counts are normalized to per million counts. TSS: Transcription

start site; TES: Transcription end site; A: Splicing acceptor site; D: Splicing donor site. The principal component analysis (PCA) of global 5hmC profiles showed no significant correlations between 5hmC and potential confounders such as sex (B) and age (C). The heatmaps show genes with differential 5hmC ($P < 0.05$) detected between (D) Patients with DN and CTRL and (E) Patients with DN and non-DN patients.

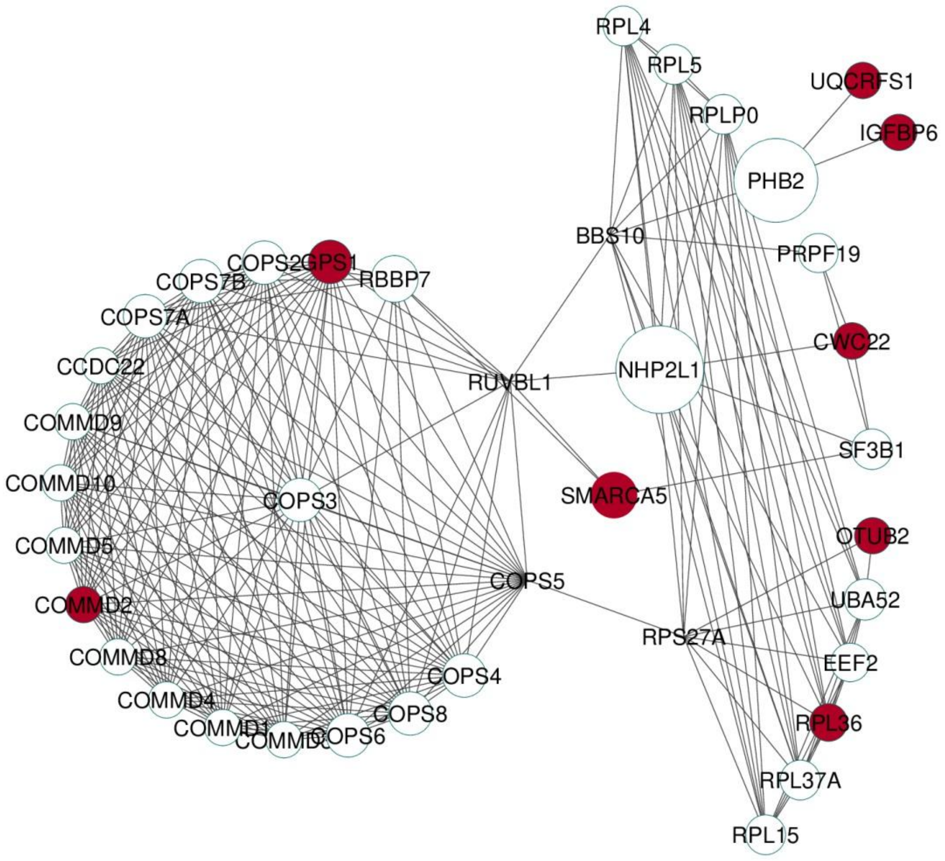

Supplementary Figure 2. PPI networks of differentially modified genes between patients with DN and controls. PPI networks were constructed based on the measurement of betweenness centrality across differentially modified gene bodies between patients with DN and CTRL. The node size is proportional to the betweenness centrality calculated from the network analysis. Differentially modified

genes are annotated as red circles, while linker genes are annotated as white circles. PPI: Protein-protein interaction; DN: Diabetic nephropathy; CTRL: Controls.

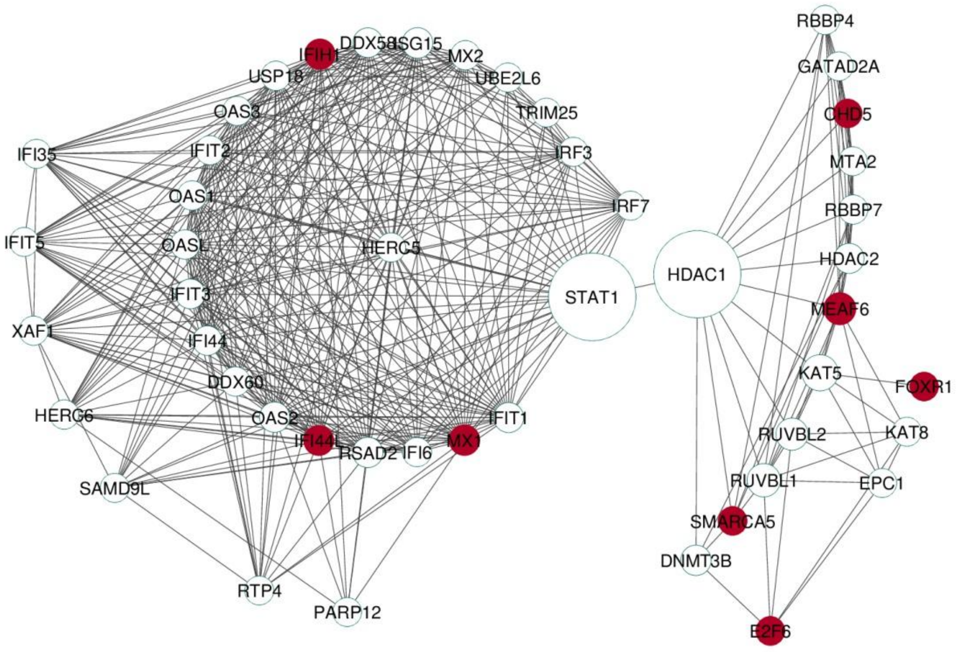

Supplementary Figure 3. PPI networks of differentially modified genes between patients with DN and non-DN. PPI networks were constructed based on the measurement of betweenness centrality across differentially modified gene bodies

between patients with DN and non-DN. The node size is proportional to the betweenness centrality calculated from the network analysis. Differentially modified genes are annotated as red circles, while linker genes are annotated as white circles. PPI: Protein-protein interaction; DN: Diabetic nephropathy.
